# Utilisation of a community-based health facility in a low-income urban community in Ibadan, Nigeria

**DOI:** 10.4102/phcfm.v7i1.735

**Published:** 2015-05-05

**Authors:** Ayodeji M. Adebayo, Michael C. Asuzu

**Affiliations:** 1 Department of Preventive Medicine and Primary Care, College of Medicine, University of Ibadan, Ibadan

## Abstract

**Background:**

Primary healthcare is established to ensure that people have access to health services through facilities located in their community. However, utilisation of health facilities in Nigeria remains low in many communities.

**Aim:**

To assess the utilisation of community-based health facility (CBHF) amongst adults in Ibadan, Nigeria

**Settings:**

A low-income community in Ibadan North West Local Government Area of Oyo State.

**Methods:**

A cross-sectional survey was conducted using a simple random sampling technique to select one adult per household in all 586 houses in the community. A semi-structured interviewer-administered questionnaire was used to collect information on respondents' sociodemographic characteristics, knowledge and utilisation of the CBHF. Data analysis included descriptive statistics and association testing using the Chi-square test at *p* = 0.05.

**Results:**

The mean age of the respondents was 46.5 ± 16.0 years; 46.0% were men and 81.0% married; 26% had no formal education and 38.0% had secondary-level education and above; traders constituted 52.0% of the sample; and 85.2% were of low socioeconomic standing; 90% had patronised the CBHF. The main reasons for non-utilisation were preference for general hospitals (13.8%) and self-medication (12.1%). Respondents who had secondary education and above, were in a higher socioeconomic class, who had good knowledge of the facility and were satisfied with care, utilised the CBHF three months significantly more than their counterparts prior to the study (*p* < 0.05). However, only satisfaction with care was found to be a significant predictor of utilisation of the CBHF.

**Conclusion:**

The utilisation of the CBHF amongst adults in the study setting is high, driven mostly by satisfaction with the care received previously. Self-medication, promoted by uncontrolled access to drugs through pharmacies and patent medicine stores, threatens this high utilisation.

## Abstrait

**Utilisation d'un établissement communautaire de santé dans un quartier urbain à faibles revenus d'Ibadan, Nigéria.**

**Contexte:**

Les soins de santé primaire ont été établis pour permettre aux gens d'avoir accès aux services de santé dans des établissements installés dans leur communauté. Cependant, l'utilisation des équipements sanitaires au Nigeria est faible dans de nombreuses communautés.

**Objectif:**

Evaluer l'utilisation d'un établissement communautaire de santé (CBHF) par les adultes à Ibadan, Nigeria

**Situation:**

Une communauté à faibles revenues dans la Zone de Gouvernement local d'Ibadan Nord Ouest (IBNWLGA) de l'état d'Oyo.

**Méthodes:**

On a effectué une enquête transversale au moyen d'une technique d' échantillonnage simple au hasard pour sélectionner un adulte par ménage dans les 586 maisons de la communauté. On s'est servi d'un questionnaire semi-structuré administré par l'interviewer pour collecter les informations sur les caractéristiques sociodémographiques, les connaissances et l'utilisation du CBHF par les personnes interrogées. L'analyse des données comprenait des statistiques descriptives et des tests d'association au moyen du test de Chi-carré à *p* = 0.05.

**Résultats:**

L'âge moyen des personnes interrogées était de 46.5 ± 16.0 ans; 46.0% étaient des hommes et 81.0% étaient mariés. Vingt-six pour cent n'avaient pas d'éducation formelle; et 38.0% avaient fait des études secondaires et plus. Les commerçants constituaient 52.0% de l'échantillon; et 85.2% avaient un statut socioéconomique modeste. Quatre-vingt-dix pour cent avaient fréquenté le CBHF. Les raisons principales pour la non utilisation de l'établissement étaient une préférence pour les hôpitaux généraux (13.8%) et l'automédication (12.1%). Les personnes interrogées qui avaient fait des études secondaires et plus, appartenaient à une classe sociale supérieure; ils connaissaient bien l'établissement et étaient satisfaits des soins, et ils avaient considérablement utilisé le CBHF trois mois avant l'enquête par rapport à leurs homologues (*p* < 0.05). Cependant, seule la satisfaction des soins était un indice significatif de l'utilisation du CBHF.

**Conclusion:**

L'utilisation du CBHF par les adultes de l'étude est élevée, et liée essentiellement à la satisfaction des soins reçus au paravent. L'automédication, encouragée par l'accès incontrôlé aux médicaments des pharmacies et des magasins qui vendent des médicaments brevetés, menace l'utilisation élevée de l'établissement.

## Introduction

Primary healthcare (PHC) is defined as:

essential healthcare based on practical, scientifically-sound and socially-acceptable methods and technology made universally accessible to individuals and families in the community by means acceptable to them at a cost that the community and country can afford to maintain at every stage of their development in a spirit of self-reliance and self-determination.^1^

It forms an integral part of both the countries' health system of which it is the central function and the main focus of the overall social and economic development of the community. It is also the first level of contact of individuals, families and communities with the national health system, bringing healthcare as close as possible to where people live and work and constitutes the first element of continuing health care process.^1^

Emphasis in healthcare has changed from healthcare *for* the people to healthcare *by* the people. Health is meant to be earned and maintained by the individuals.^2^ However, despite the fact that it is a fundamental human right, the community also has a role to play in contributing to the health of its members and the community as a whole. Apart from contributing toward the planning and implementation of planned programmes aimed at developing the community, the community also needs to utilise relevant health facilities in an appropriate manner.

The inception of PHC has facilitated some communities to have at least one health care facility sited as close as possible to where they live or work in all the districts or local government areas of many of the states in Nigeria. However, siting of health care facility does not necessarily translate to its utilisation; more so, that one of the major factors maintaining high mortality rate in Nigeria is poor access to and utilisation of health services.^3,4^ Several other factors, such as availability and cost of services, location of facility from clients/patients, competencies and attitudes of service providers, the peculiarity of patients' need and adequacy of resources can affect effective healthcare delivery. In a literature review of the situation of health-seeking behaviour in developing countries, by Shaikh and Hatcher in Pakistan,^5^ physical, socioeconomic, cultural and political contexts were documented as being the factors responsible for utilisation of a healthcare system, whether public or private, formal or informal.^5^ Details of these factors may include sociodemographics, social structures, educational status, gender discrimination, cultural beliefs and practices, employment status, women status, political will, the disease pattern, environmental conditions and healthcare system itself.^5^

Primary healthcare is established to ensure that people have access to health services through health facilities located in their community. However, utilisation of health facilities in Nigeria remains unacceptably low in many communities. A household survey amongst 630 respondents in Northern Nigeria showed that the majority preferred to use patent medicine stores (53.63%) compared with only 7.6% who utilised the PHC services.^6^ In another study to assess the utilisation of PHC facilities in a rural community in south-western Nigeria, 44% of the respondents who were ill in the three months preceding the survey had used the health facility.^7^ In many, if not all countries in Africa, as well as in other developing countries worldwide, most morbidities are treated at home and are never reported to the formal healthcare system.^8^ Clinical problems that present to the formal healthcare facilities are, therefore, only the tip of the iceberg. For example, for every case of febrile illness attended to in the formal healthcare facilities, approximately 4–5 more existed in the community in resource-poor countries.^8^

Studies on health services utilisation often seek to understand both the frequency and trends in the use of health services, as well as the possible mechanisms that may be associated with the pattern of use.^9^ Good knowledge and understanding of utilisation also help health providers and health systems managers to plan and improve on health services. However, it has been observed that such studies have been neglected, especially since the early 1990s. Given that most PHC facilities in Nigeria were established without an evaluation of their accessibility to the communities they are meant to serve, gaining an understanding of how people use health facilities cannot be over-emphasised. This is even more so in developing countries, including Nigeria, where people's knowledge of and attitude toward health services and the use of these services are still poor.^4,10,11,12^

### Aims and objectives

Given that the utilisation of the CBHF by the community members at Idikan, Ibadan, Nigeria has hitherto not been assessed since its advent, the current study aimed to assess the utilisation of the facility within the three months preceding the study and to identify reasons for any lack of utilisation. It is hoped that the findings of this study will inform interventions for the improvement of PHC services in the community, hence the need for this study. Achieving this will enable better decisions to be made, which should result in better, more effective primary care for the people of the area in the long run. Therefore, the objective of this study was to assess the utilisation of the facility within the three months preceding the interview as well as to assess any reasons for any lack of utilisation of the facility, so that these can be corrected, if possible, for the improvement of PHC in the area.

## Research methods and design

### Study design

This was a community-based descriptive cross-sectional survey.

### Research setting

The study was conducted in Idikan, a low-income urban community located in Ibadan North West Local Government Area of Oyo State, South-western Nigeria. The community has an estimated population of 13 902 based on the 2006 population census.^13^ The majority of the inhabitants are petty traders, artisans and farmers – the men are mostly artisans and farmers whilst the women are traders. Almost everybody in the community is from the Yoruba tribe – the dominant ethnic group in the south-western part of Nigeria. A great majority of the inhabitants have no formal education and belong to a low socioeconomic class. Idikan, as with other traditional areas of Ibadan, is grossly unplanned and has limited access to pipe-borne water and an irregular electricity supply. The CBHF was established in 1963 to serve the urban Idikan community. The programme was designed to promote health and to provide basic medical services to the community members as well as to provide an avenue for teaching urban community healthcare to the students of the Ibadan Medical School. With the advent of PHC in 1978, the service was expected to provide as much of all community/PHC services to the community as possible. The services are run from Monday to Friday for six hours per day by community health nurses and resident doctors, supervised by consultant community physicians from the Department of Community Medicine, University College Hospital, Ibadan. Two members of the community (community health assistance) provide assistance in the running of services at the facility. Currently, the clinic attends to an average 15–20 adults per day. There is a community health committee meant to ensure community participation in sustaining the services provided at this facility. The members of the committee contribute to the planning and implementation of programmes for their development through provision of funds, logistic and human resources; and in the utilisation of this facility.

### Study population

The study population consisted of male and female adults residing in selected households.

### Sample size calculation

The sample size was calculated using Leslie Kish's formula for descriptive surveys.^14^ A minimum sample size of 398 respondents was estimated, taking into consideration the prevalence of utilisation of health facilities of 44% from a previous study,^15^ with a critical ratio of 1.96, a level of precision of 5% and a non-response rate of 5%.

### Sampling methods

There were a total of 586 houses in Idikan community with an average of 3–4 households per dwelling. All 586 houses were visited. In places where there were more than one household per house, one household was selected randomly by balloting. In the selected household, one adult was selected by a simple random sampling technique by balloting.

### Research instrument and data collection

The study was conducted using a semi-structured interviewer-administered questionnaire. Five research assistants were recruited from the community and trained in order to administer the questionnaire. The interview was conducted mostly in the evening, which is when most of the community members were around. The questionnaire covered: sociodemographic characteristics; perception of community health services by adult members in the community; pattern of presentation in the three months preceding the interview; the utilisation of the CBHF for common illnesses in the three months preceding the interview; and obstacles against use of the community health services.

The questionnaire was standardised after it had been critiqued during a departmental proposal presentation with consultants, senior registrars and registrars present. Through constructive criticism, any possible shortcomings which could affect the quality and feasibility of the study were identified and rectified. The questionnaire was translated into Yoruba for the benefit of the majority who were predominantly Yoruba speaking, then translated back to formal English by a different translator from the first to ensure that there was no error in translation and that the original meaning was retained.

Pretesting was carried out on 15 subjects in Abebi, an adjoining community in the area which was not part of the study area but has similar characteristics. The pre-test was found necessary in order to ensure clarity of interpretation, ease of completion, reduce respondents' bias and generate useful questions not initially conceived but very germane to the quality of the study and to correct any ambiguity whatsoever detected. Corrections and relevant restructuring were made in places of ambiguity.

### Data collation and analysis

The data obtained were sorted out, edited and manually cleaned and recoded where necessary. Data were entered into the computer and analysed with SPSS software v 16.0 (SPSS Inc., Chicago, IL 2007). Data analysis was done with the assistance of a statistician using both descriptive and inferential statistics. Descriptive statistics such as percentages or proportions were used to describe the qualitative or categorical variables. The Chi-square test was used to examine the relationship between two categorical variables. The test was carried out at 5% level of significance.

Responses to questions on knowledge of the CBHF were converted to a 40-point score by coding a correct answer as ‘1' and a wrong answer as ‘0'. The knowledge scores were generated giving minimum and maximum obtainable scores of 0 and 40 respectively. Respondents were categorised into having good, fair and poor knowledge using 75% and above, 50% – 74% and 49% and below of the maximum obtainable scores, respectively. Seventy-five per cent was used as the lowest limit for good knowledge because the CBHF had been in Idikan community for more than two decades before this assessment. It was located both centrally and within 1 km of all the community members for easy accessibility. Hence, respondents with a knowledge score of 30 and above were reported as having good knowledge, fair (20–29) and poor (< 20). Similarly, responses to perception questions were converted to a 60-point score. Perception scores were generated with minimum and maximum obtainable scores of 12 and 60 respectively. Respondents were classified into having poor, fair and good perception of some characteristics of the CBHF using 75% and above, 50% – 74% and 49% and below of the total obtainable scores, respectively. Hence, those with scores of 36 or less were regarded as having poor perception, 37–49 fair and ≥ 50 good perception. Satisfaction with the services at the CBHF was assessed by asking if respondents were satisfied with the services received at the facility, generating a ‘yes' or ‘no' response.

Multivariate analysis using binary logistic regression^16^ was used to identify predictors of utilisation of the CBHF. The independent variables entered into the logistic regression model were those that were significant at 10% (*p* < 0.1) on bivariate analysis.

The occupation of respondents was classified into high and low occupational class for ease of bivariate analysis, in some instances by modifying the social class based on occupation alone, as adopted from Rose and Pevalin (2001).^17^ Those classified as high occupational class were those in social class I and II (including professionals, senior civil servants and those in managerial occupations), whilst the low occupational class consisted of those in social class III, IV and V (including traders, artisans, farmers, drivers, etc.). Socioeconomic status was classified into high and low using the educational level and occupation of respondents. Those in the high occupational class with tertiary education were classified as the high socioeconomic class whilst those in the low occupational class with secondary education and below were classified as the low socioeconomic class.

### Ethical considerations

Ethical approval was obtained from Oyo State Ethical Review Committee, State Ministry of Health (reference number AD/13/479/146). Permission and cooperation were sought from the High Chief of Idikan Community. Verbal informed consent was also ensured from all the participants. No names were recorded on the questionnaire so as to ensure confidentiality; and codes were used for identification of respondents instead of names.

## Results

A total of 586 households were visited, from which 554 respondents consented – a response rate of 95%. The respondents' sociodemographic characteristics are shown in Table 1. The mean age was 46.5 ± 16 years with the highest proportion (*n* = 237, 42.6%) being in the 30–49 year age group. Most were women (*n* = 300, 54%) and from a monogamous family setting (*n* = 352, 63.5%). Traders comprised 52.2% (*n* = 289) of the sample, 173 (31.2%) had secondary education and 347 (62.2%) were of the Islamic religion. Most of the respondents were married (*n* = 447, 80.7%), of the Yoruba tribe (*n* = 507, 91.5%) and within the low socioeconomic status (*n* = 472, 85.2%).

**TABLE 1:** Sociodemographic characteristics of respondents.

Variables (*N* = 554)	Frequency	Percentage
**Gender**		
Male	254	46
Female	300	54
**Age group**		
< 20	4	0.7
20–29	78	14.1
30–39	121	21.8
40–49	116	20.8
50–59	87	15.7
60–69	74	13.4
> 70	57	10.3
No response	17	3.1
**Ethnicity**		
Yoruba	507	91.5
Igbo	43	7.8
Hausa	4	0.7
**Marital status**		
Single	29	5.2
Married	447	80.7
Separated	13	2.3
Divorced	13	2.3
Widowed	52	9.4
**Religion**		
Islam	347	62.6
Christianity	205	37
Traditional	2	0.4
**Type of marriage**		
Monogamous	352	63.5
Polygamous	202	36.5
**Level of education**		
No formal education	143	25.8
Quranic	49	8.8
Primary	153	27.6
Secondary	173	31.2
Tertiary	36	6.5
**Occupation**		
Professional†	24	4.3
Civil servant	25	4.5
Artisan	90	16.2
Trading	289	52.2
Unemployed	56	10.1
Other‡	70	12.6
**Monthly income[Naira]**		
< 5000	241	43.5
5000–9999	79	14.3
> 10 000	124	22.3
No response	110	19.9
**Socioeconomic status**		
Low (1–5)	472	85.2
High (6–9)	82	14.8
**Distance of respondents house from health facility**		
≤ 5 km	434	78.3
> 5 km	120	21.7

†, Engineers, teachers; ‡, Clergy, students, drivers, farmers.

The majority (*n* = 484, 82.5%) of the respondents felt that the best place to seek help when sick is the CBHF. Regarding the service components of PHC available at the CBHF, 211 (42.5%) knew about health education and 183 (36.9%) were aware of referral services. This was followed by knowledge of antenatal services (*n* = 169, 34.1%) and immunisation (*n* = 118, 23.8%). Questions were asked to test the respondents' knowledge about some of the characteristics of the services provided at the CBHF, for example: if they were provided free; rendered 24 hours daily; availability of a community health committee; and if members of the community are part of the working staff. In each of the first three knowledge questions, well above 50% (*n* = 336, 67.7%; *n* = 361, 72.8%; and *n* = 310, 62.5%, respectively) answered correctly, except for the last question (if members of the community are part of the working staff), where only 47.6% were correct. Just over half of the respondents (*n* = 284, 57.3%) had good knowledge of the administrative structure and functions of the CBHF as well as the services there (Table 2).

**TABLE 2:** Distribution of respondents regarding knowledge of the community-based health facility.

Knowledge areas (*N* = 496)	Correct *n* (%)	Incorrect *n* (%)
**Owner of the facility**	373 (75.2)	123 (24.8)
**Types of services provided**		
Antenatal care	327 (65.9)	169 (34.1)
Immunisation	378 (68.2)	118 (23.8)
Family planning	454 (91.5)	42 (8.5)
Treatment of common ailments	389 (78.4)	107 (21.6)
Health education	285 (57.5)	211 (42.5)
Referral	313 (61.3)	183 (36.9)
Ultra Sound Scan	298 (60.1)	198 (39.9)
**Characteristics of services provided**		
Free	336 (67.7)	160 (32.3)
24 hours	361 (72.8)	135 (27.2)
**Availability of community health committee**	310 (62.5)	186 (37.5)
**Members of the community provide assistance at the facility**	236 (47.6)	260 (52.4)
**Overall knowledge rating regarding the community-based health facility**		
Good (30 and above)	284	57.3
Fair (20-29)	116	23.4
Poor (19 and below)	96	19.3

**FIGURE 1:**
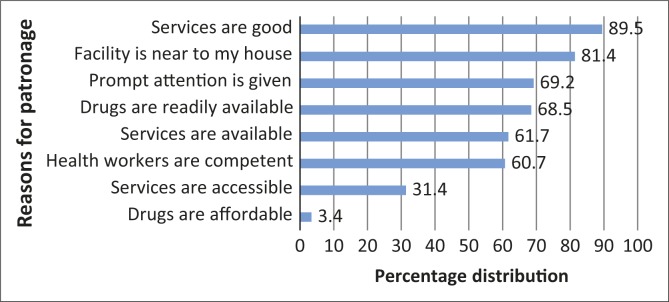
Percentage distribution of respondents by reasons for utilising the community-based health facility.

Most the respondents (*n* = 496, 89.5%) had patronised the CBHF. Of the 554 respondents, 369 (76.1%) reported that they were satisfied with the care received at the CBHF. Amongst those that had ever received treatment in the facility, the perceived reasons for seeking care include good services (89.5%), nearness to the house (84.1%), prompt attention (69.2%) and available of essential drugs (68.5%) (Figure 1). Amongst those that had never utilised the CBHF, 21 (36.2%) proffered reasons for non-patronage as preference for general hospital (13.8%), self-medication (12.1%) and one person (1.7%) reported a preference for traditional practitioners.

Amongst those that sought medical care outside the home in the three months preceding the interview, 212 (93.8%) specified their sources of care. One hundred and fifty-one respondents (66.8%) reported the CBHF as their source of medical care three months prior to interview, 34 (15.0%) a private hospital, 16 (7.1%) government-owned hospitals and 11 (4.9%) a faith-based organisation.

Results of the bivariate analysis are shown in Table 3, indicating that the higher occupational class (*p* = 0.013), higher socioeconomic class (*p* = 0.01), having secondary education and above (*p* = 0.023), being unmarried (*p* = 0.002), satisfaction with previous care (*p* < 0.001) and having good attitude, perceptions and knowledge (*p* < 0.001) were all associated with having received treatment from the CBHF in the preceding three months.

**TABLE 3:** Association between utilisation of services at the community-based health facility for three months before survey and respondents' characteristics.

Variables	Received treatment in last 3 months		*X^2^*	*p*-value
	Yes *n* (%)	No *n* (%)		
**Age group (yrs)**				
< 30	21 (25.6)	61 (74.4)	3.938	0.140
30–59	96 (29.6)	228 (70.4)		
60 and above	27 (20.6)	104 (79.4)		
**Gender**				
Male	72 (28.2)	183 (71.8)	0.228	0.633
Female	79 (26.4)	220 (73.6)		
**Occupational class**				
High	18 (43.9)	23 (56.1)	6.188	0.013*
Low	133 (25.9)	380 (74.1)		
**Educational level**				
No formal	41 (21.4)	151 (78.6)	7.569	0.023*
Primary	40 (26.1)	113 (73.9)		
Secondary and above	70 (33.5)	139 (66.5)		
**Socioeconomic status**				
Low	119 (25.2)	353 (74.8)	6.722	0.010*
High	32 (39.0)	50 (61.0)		
**Occupation of head of household**				
Professional	13 (43.3)	17 (56.7)	5.130	0.275
Civil servant	9 (28.1)	23 (71.9)		
Artisan	24 (23.3)	79 (76.7)		
Traders	69 (28.0)	177 (72.0)		
Other	36 (25.2)	107 (74.8)		
**Marital status**				
Never	15 (51.7)	14 (48.3)	9.24	0.002*
Ever	136 (25.9)	389 (74.1)		
**Ethnicity**				
Yoruba	139 (27.4)	368 (72.6)	0.077	0.781
Other	12 (25.5)	35 (74.5)		
**Religion**				
Christianity	62 (30.2)	143 (69.8)	1.465	0.226
Islam	89 (25.5)	260 (74.5)		
**Family type**				
Polygamous	92 (26.1)	260 (73.9)	0.611	0.435
Monogamous	59 (29.2)	143 (70.8)		
**Household income**				
< 5000	71 (31.4)	157 (68.6)	1.424	0.491
5000–9000	23 (25.3)	68 (74.7)		
10 000 and above	34 (27.4)	90 (72.6)		
**Satisfaction**				
Yes	136 (36.9)	233 (63.1)	37.948	<0.001*
No	8 (6.9)	108 (93.1)		
**Distance**				
≤ 5	113 (26.0)	321 (74.0)	1.503	0.220
> 5	38 (31.7)	82 (68.3)		
**No of days sickness lasted**				
≤ 14 days	136 (30.8)	306 (69.2)	0.001	0.982
> 14 days	15 (30.6)	34 (69.4)		
**Severity**				
Mild	104 (31.1)	230 (68.9)	0.033	0.856
Severe	47 (30.3)	108 (69.7)		
**Knowledge**				
Poor (< 20)	6 (6.3)	90 (93.8)	43.269	< 0.001*
Fair (21–29)	26 (22.4)	90 (77.6)		
Good (30 and above)	114 (40.1)	170 (59.9)		
**Perception**				
Poor	5 (5.3)	90 (94.7)	33.421	< 0.001*
Fair	12 (40.0)	18 (60.0)		
Good	129 (34.8)	242 (65.2)		

* *p*-values < 0.05

In the multivariate logistic regression analysis (Table 4), only satisfaction with care received was a significant predictor of utilisation of services at the CBHF. Respondents that were not satisfied with care were less likely (OR = 0.378; 95% CI = 0.144–0.994; *p* = 0.049) to use the CBHF.

**TABLE 4:** Adjusted odds ratio of predictors of utilisation of the community-based health facility.

Variables	Odds ratio	95% CI	*p*-value
**Occupational class**			
High	1.000		
Low	0.594	0.203–1.737	0.342
**Level of education**			
No formal	1.000		
Primary	0.904	0.520–1.571	0.719
Secondary	0.913	0.514–1.621	0.756
**Socioeconomic status**			
Low	1.000		
High	1.437	0.615–3.353	0.402
**Marital status**			
Never	1.000		
Ever	0.409	0.138–1.213	0.107
**Satisfaction**			
Yes	1.000		
No	0.378	0.144–0.984	0.049*
**Knowledge**			
Poor	1.000		
Fair	0.831	0.132–5.249	0.844
Good	1.711	0.276–10.618	0.564
**Perception**			
Poor	1.000		
Fair	4.278	0.495–36.982	0.187
Good	3.326	0.448–24.682	0.240

* *p*-value < 0.05

## Discussion

This study was carried out to assess the utilisation of the CBHF by adult members in a low-income urban community in Ibadan for the purpose of promoting/optimising and upgrading PHC in the area. The majority of the respondents (89.5%) had utilised the CBHF at one time or the other and utilisation of services in the preceding three months was equally high (89%). Utilisation in the preceding three months was in relation to treatment of injuries and ailments, whether acute or chronic. However, the respondents' awareness of the service components of PHC provided at the CBHF was low. Less than half knew about health education, 36.9% were aware of referral services, followed by antenatal services (34.1%) and immunisation (23.8%). This means that respondents were not aware of the full range of the services provided at the CBHF based on the PHC service components. Utilisation of services in the preceding three months showed that they were more familiar with use of the facility for treatment of disease conditions such as malaria, hypertension and diabetes. The low awareness of the service components of PHC amongst respondents in this study may be a result of poor enlightenment of the public regarding the component services available at the centre with health workers, provision of services for only six hours without admission; non-provision of delivery services, in addition to antenatal care, which would have encouraged patients and relatives to stay for a longer period in the facility and provide opportunities for exposure to the other services being offered at the centre. The poor awareness reported in our study is in contrast to a community-based study in India where awareness of services was high but utilisation was relatively low (54.9%).^12^ Whilst our study assessed the various service components of PHC, the Indian study only inquired whether respondents were aware of the availability of PHC without exploring awareness of the various service components on offer. The relatively low awareness of the various services available at the CBHF, as documented by this study, will cause under-utilisation of the available services. Efforts at creating awareness of the available component services of PHC and promoting community mobilisation with the support of the community members will further benefit the community members by enhancing their use of the available promotive and preventive services at this facility.

The reasons adduced for utilisation of the CBHF were that services were good and readily available, health facility was nearer to their homes, drugs were readily available and attention was prompt. Reasons for non-utilisation in this study included preference for general hospitals, self-medication, private hospitals and traditional healers. In a study to determine utilisation of approved health facilities for delivery in Ile-Ife, the reasons given for non-utilisation of the health facilities at hand were time of onset of labour, problems with transport, fear of surgery, husband and/or family influence and the fact that delivery was assisted by Traditional Birth Attendants (TBAs) and relatives.^15^ Even though our study assessed services in relation to various service components of PHC compared with the Ile-Ife study, which was specifically in facilities for delivery services, the different hours of service are not comparable: six hours in our facility as opposed to 24 hours in Ile-Ife. Reasons for non-utilisation in India were ‘faith in quacks, inconvenient timing of the primary health centre, long queues, non-availability of drugs, and investigations'.^12^ Interventions to address these factors will improve the current utilisation rate.

The practice of self-medication amongst 12.1% of the respondents is worthy of mention. Considering the fact less than a third reported that the services at the CBHF were not readily accessible may be explained by the fact that services are only accessible in this facility for a maximum of six hours per day. This may force community members to become more dependent on self-medication and traditional healers because drugs are not available at all hours. This automatically makes the services of ‘quacks’ and patent medicine merchants readily available. However, there is also the possibility that their low level of education and strong cultural beliefs could contribute equally to this. However, a survey in Kathmandu, Nepal, showed that individuals turned to modern health sector ‘because they are dissatisfied with the previous folk or traditional professional consultation or because or traditional practitioners had advised them to seek modern hospital services’.^18^ Another study in South Africa on community knowledge and perception of malaria reported that 66.9% of the respondents would seek treatment at the hospital when symptoms are severe.^19^ From a study conducted in the former Soviet Union on health service utilisation, the main reason for not seeking care was lack of funds to pay for treatment (42.5%), self-treatment with home remedies (32.9%) and purchase of non-prescription medication.^20^ An extension of the hours of services at the CBHF may reduce clients' exposure to use of self-medication and traditional healers. This will prevent the clinical and policy implications of buying over-the-canter drugs which may generate drug resistance, increased morbidity and mortality through incompletely-treated cases, use of wrong medication, sub-normal doses and the adverse effects of drugs and herbal medications.

The findings of this research on bivariate analysis indicated that utilisation patterns can be explained, to a large extent, by factors relating to occupational class, socioeconomic status, level of education and level of satisfaction, as well as knowledge of and perception of respondents toward the services. However, it was only the level of satisfaction that was a significant predictor on logistic regression. These findings are consistent with findings from prior research: a bivariate analysis in determinants of utilisation of health services in the western states of Nigeria revealed age, level of education, type of education, place of work and attitudes toward services as being significant factors.^21^ A similar study in the determinants of maternal services in a rural Nigeria city showed that the mother's education and occupation of, as well as the husband's religion and occupation were associated significantly with delivery at a health facility on logistic regression analysis. This study showed a higher likelihood of utilisation of health services amongst post-primary education mothers than those with no occupation; and in Christian mothers versus their counterparts.^22^ ‘The woman's educational level and total number of living children were the most significant predictors of prenatal care utilization' in Vietman.^23^ In another study on utilisation of an approved health facility for delivery in Ile-Ife, south west Nigeria, ‘educational status of the mother, religious beliefs, distance from approved health facilities more than 5 km and attitude of health workers were amongst the factors significantly influencing choice of place of delivery' by the mother.^15^ This shows that promoting most of these factors through female education and positive attitudes of health workers toward work will further enhance the patronage of the CBHF. The results from both bivariate and multivariate analyses confirmed the importance of a mother's education in explaining the utilisation of maternal health services in a study to determine the use of such services in rural Bangladesh. The Bangladesh study also reported the significant effect of severity of disease condition in predicting the utilisation of maternal health care. Multivariate analysis indicated that women having had a life-threatening condition were more likely to seek care from a health facility.^24^

‘The distance patients must travel in order to obtain treatment has long been recognized as a primary determinant of the utilization of health care facilities’.^25^ However, it was not found to be a significant factor in this study, contrary to the findings of Esimai et al.^15^ and Chakraborty et al.^24^ The distance is especially significant in:

[*the*] Third World settings where the density of Western type health facilities is often low, where the majority of patients are likely to make the journey for treatment as pedestrians and where there are viable and usually more accessible alternate sources of medicine.^25^

## Conclusion

The utilisation of the CBHF at Idikan was high but the awareness of the various service components of PHC was low. Higher occupational and socioeconomic status, higher level of education, satisfaction with previous care, good awareness and perception of the CBHF were factors associated with utilisation of CBHF, although only satisfaction with previous care predicted utilisation on logistic regression.

### Implications or recommendations

Information on healthcare utilisation has important policy implications in health systems development. Public awareness programmes that mobilise the community to participate in the design and running of the CBHF need to be developed in order to increase awareness and sustain utilisation of the services at the CBHF. Issues surrounding waiting time, availability of drugs and accessibility should also be addressed by the institution in order to sustain the current level of utilisation. The stakeholders should review the hours of service from the current maximum of six hours to 24 hours; this might require scaling up of resources.
